# The Bath

**Published:** 1891-04

**Authors:** 


### THE BATH.

The following instructions of the secretary to the Royal Humane society of England upon bathing, will be found of value.

Avoid bathing within two hours after a meal.

Avoid bathing when exhausted by fatigue or from any other cause.

Avoid bathing when the body is cooling after perspiration.

Avoid bathing altogether in the open air if, after having been a short time in the water, there is a sense of chilliness, with numbness of the hands and feet; but bathe when the body is warm, providing no time is lost in getting into the water.

Avoid chilling the body by sitting or standing undressed on the banks or in boats after having been in the water.

Avoid remaining too long in the water, but leave the water immediately there is the slightest feeling of chilliness. The vigorous and strong may bathe early in the morning on an empty stomach. The young and those who are weak had better bathe two or three hours after a meal; the best time for such is from two to three hours after breakfast. Those who are subject to attacks of giddiness or faintness, and those who suffer from palpitation or other sense of discomfort at the heart, should not bathe without first consulting their medical adviser.

